# The clinicopathological significance of monocarboxylate transporters in testicular germ cell tumors

**DOI:** 10.18632/oncotarget.24910

**Published:** 2018-04-17

**Authors:** Eduardo C.A. Silva, Flavio M. Cárcano, Murilo Bonatelli, Maurício G. Zaia, Filipa Morais-Santos, Fátima Baltazar, Luiz F. Lopes, Cristovam Scapulatempo-Neto, Céline Pinheiro

**Affiliations:** ^1^ Pathology Department, Barretos Cancer Hospital, Barretos, São Paulo, Brazil; ^2^ Medical Oncology Department, Barretos Cancer Hospital, Barretos, São Paulo, Brazil; ^3^ Barretos School of Health Sciences Dr. Paulo Prata – FACISB, Barretos, São Paulo, Brazil; ^4^ Molecular Oncology Research Center, Barretos Cancer Hospital, Barretos, São Paulo, Brazil; ^5^ Life and Health Sciences Research Institute (ICVS), School of Health Sciences, University of Minho, Braga, Portugal; ^6^ ICVS/3B’s-PT Government Associate Laboratory, Braga/Guimarães, Portugal; ^7^ Barretos Children's Cancer Hospital, Barretos, São Paulo, Brazil

**Keywords:** testicular neoplasms, testicular germ cell tumors, monocarboxylate transporter, immunohistochemistry, Warburg effect

## Abstract

**Background:**

Metabolic reprogramming is one of the hallmarks of cancer. The hyperglycolytic phenotype is often associated with the overexpression of metabolism-associated proteins, such as monocarboxylate transporters (MCTs). MCTs are little explored in germ cell tumors (GCTs), thus, the opportunity to understand the relevance of these metabolic markers and their chaperone CD147 in this type of tumor arises. The main aim of this study was to evaluate the expression of MCT1, MCT2, MCT4 and CD147 in testicular GCT samples and the clinicopathological significance of these metabolism related proteins.

**Results:**

MCT1, MCT4 and CD147 were associated with higher stages, higher M and N stages and histological type, while MCT4 was also associated with higher risk stratification, presence of vascular invasion, and lower overall and event free survival. MCT4 silencing in JEG-3 had no significant effect in cell viability, proliferation and death, as well as extracellular levels of glucose and lactate. However, MCT4-silenced cells showed an increase in migration and invasion.

**Conclusion:**

The proteins herein studied, with the exception of MCT2, were associated with characteristics of worse prognosis, lower global and event free survival of patients with GCTs. Also, *in vitro* MCT4 silencing stimulated cell migration and invasion.

**Materials and Methods:**

Immunohistochemical expression was evaluated on samples from 149 adult patients with testicular GCT, arranged in Tissue Microarrays (TMAs), and associated with the clinicopathological data. Also, MCT4 silencing studies using siRNA were performed in JEG-3 cells.

## INTRODUCTION

Although testicular germ cell tumors (TGCTs) are relatively rare neoplasms, they are the most frequent solid tumors affecting young males [1, 2]. Importantly, the natural history of these tumors has changed over the past three decades, thanks to platinum-based chemotherapy regimens, with current 5-year overall survival rates over 90% regardless of histological type (seminoma and nonseminomatous) and cure rates over 90% in the low risk group. Although the majority of patients with TGCTs have good responses to treatment, some of them resist to therapy and present disease progression (reviewed in [3, 4]). Therefore, it is important to better understand the biological heterogeneity of TGCTs, in order to optimize treatment by exploring new pathways involved in tumor development and progression.

Nearly all TGCTs present the isochromosome 12p, which has been pointed as the triggering event leading to invasive growth in TGCTs. However, the precise functional mechanisms involving isochromosome 12p as a player in TGCTs are yet to be described (reviewed in [5]). Despite this lack of information, other relevant biological characteristics involved in the multistep development of human tumors, as proposed by Hanahan and Weinberg [6], should be considered in the pursuit to a better understanding of the biological features of TGCTs. In this context, the reprogramming of energy metabolism, recently described as one of the hallmarks of cancer cells [6, 7], emerges as a promising candidate.

Malignant cells tend to have a hyperglycolytic phenotype, a metabolic reprogramming in which cancer cells have a preference for energy production through glycolysis, even in the presence of oxygen, a phenomenon called the Warburg effect [8, 9]. This phenotype leads to greater consumption of glucose and produces more lactate than the normal metabolic profile, which depends mostly on oxidative phosphorylation [6–9]. To avoid intracellular acidification due to this altered metabolism, some proteins are upregulated, including monocarboxylate transporters (MCTs), which are also responsible for lactate transport [10]. MCTs belong to a family that comprises 14 different isoforms, from which only MCT1-4 have been described as lactate transporters: isoforms 1 and 4 are mainly associated with lactate efflux and their main chaperone is CD147 [11–13], MCT2 is involved in lactate influx and its chaperone is gp70 [14, 15]. In fact, many different types of cancer overexpress MCT1, MCT4 and CD147, and this overexpression is associated with worse prognosis [16–24]. As pointed out by many studies, these proteins may play a relevant role as prognostic markers and be explored as possible therapeutic targets [24–31].

Although studies evaluating MCTs in TGCTs are lacking, there is evidence that these tumors are highly glycolytic, as they overexpress glucose transporter 3 (GLUT3) [32, 33], a protein often expressed by malignant neoplasms [33, 34], and, importantly, their high glucose consumption rates are demonstrated by ^18^F-Fluorodeoxyglucose positron emission tomography studies [35–37].

Since MCTs need to be more explored, including in TGCTs, it is important to evaluate the relevance of these metabolic markers and the chaperone CD147 in this type of tumor. Therefore, the aims of this study were to evaluate the expression of MCT1, MCT2, MCT4 and CD147 in normal tissues and TGCTs, using tissue microarrays (TMAs), to validate the TMA method for the study of these proteins in a heterogeneous type of tumor, to compare the expression in tumors with normal tissue, to correlate the expression with clinicopathological data, and to further explore the contribution of MCTs for tumor aggressiveness using GCT *in vitro* models.

## RESULTS

### Immunohistochemistry expression and cutoffs

The immunohistochemical expression was graduated in extension (percent of positive cells) and intensity (negative/weak/moderate/intense). Cutoffs for positivity of each marker were defined based on the area under the ROC curve, considering the sum of scores and the occurence of clinical event (recurrence, progression or death). For MCT1 and MCT4, the score with best sensitivity and specificity was 6, while for MCT2 it was 3 and for CD147 it was 5 (Table 1).

**Table 1:** Markers’ cutoffs based on the area under the ROC curve, and their classification measures

Marker	Cutoff	Sensitivity (%)	Specificity (%)	PPV	NPV	Area under ROC curve (CI)
MCT1	≥6	64.0	53.8	22.5	87.7	0.597 (0.479–0.715)
MCT2	≥3	14.3	84.4	16.7	81.8	0.509 (0.264–0.754)
MCT4	≥6	69.6	55.1	23.2	90.3	0.647 (0.537–0.758)
CD147	≥5	35.0	77.5	25.9	84.1	0.557 (0.416–0.698)

CI – Confidence interval; PPV – Positive predictive value; NPV – Negative predictive value.

**Figure 1:**
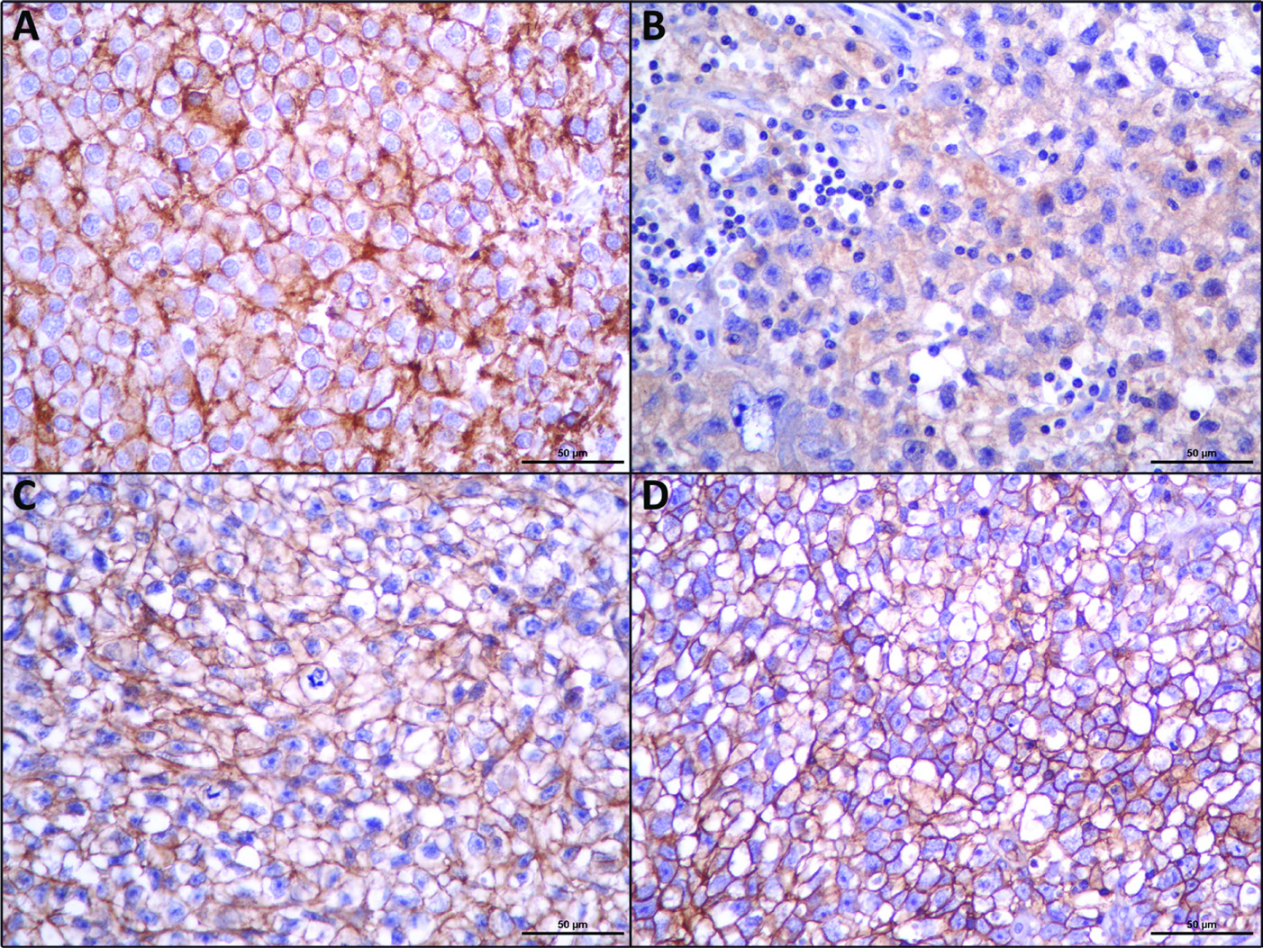
Immunohistochemical expression of MCT1 (**A**), MCT2 (**B**), MCT4 (**C**), CD147 (**D**), in seminomas. MCT2 shows cytoplasmic staining while the other proteins show an evident plasma membrane expression.

As can be observed in Figure 1, MCT1, MCT4 and CD147 were observed at the plasma membrane, while MCT2 was mostly detected only at the cytoplasm.

### Validation of TMA method

Analysis of agreement between TMA and whole tissue sections showed an accuracy for TMA method of 79.3% for MCT1, 100% for MCT2, 76.6% for MCT4 and 79.3% for CD147.

### Clinicopathological significance of MCTs and CD147

**Figure 2:**
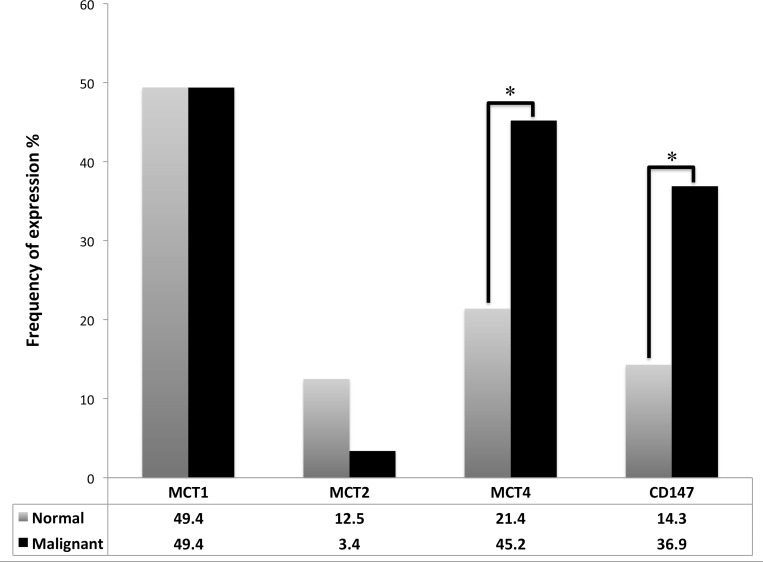
Frequency of plasma membrane expression of MCT1, MCT2, MCT4 and CD147 in TGCTs and normal testicular tissue McNemar's test was used to assess differences of expression frequency between tumor and normal tissue. ^*^p = 0.001.

When compared to paired normal tissue samples (Figure 2), TGCTs significantly overexpressed MCT4 (*p =* 0.001) and CD147 (*p =* 0.001).

Table 2 shows the associations between the expression of the metabolism related proteins and the clinicopathological parameters of the patients. MCT1 expression showed a statistically significant association with higher N stages (*p* = 0.015), higher M stage (*p* = 0.002), stages higher than I (*p* = 0.001), and nonseminomatous histology (*p <* 0.001). MCT4 was associated with higher T stages (*p* = 0.004), higher M stage (*p* = 0.037), stages higher than I (*p* = 0.045), presence of vascular invasion (*p* = 0.002), nonseminomatous histology (*p <* 0.001), and higher International Germ Cell Cancer Collaborative Group (IGCCG) risk stratifications (*p* = 0.030). CD147 was associated with stages higher than I (*p* = 0.003), higher N stages (*p* = 0.022), higher M stage (*p* = 0.020), and nonseminomatous histology (*p <* 0.001). In opposition, MCT2 showed no association with the clinicopathological data.

**Table 2:** Association of MCT1, MCT2, MCT4 and CD147 expression with clinicopathological parameters

	MCT1		MCT2			MCT4			CD147			
	*n*	Positive (%)	*p*	*n*	Positive (%)	*p*	*n*	Positive (%)	*p*	*n*	Positive (%)	*p*
**T stage**			0.148			0.081^*^			**0.004**			0.069^*^
T1	**85**	35 (41.2)		**85**	5 (5.9)		**83**	29 (34.9)		**82**	25 (30.5)	
T2+T3+T4	**58**	31 (53.4)		**58**	0 (0.0)		**57**	34 (59.6)		**57**	26 (45.6)	
**N stage**			**0.015**			1.000^*^			0.094			0.022^*^
N0	**84**	33 (39.3)		**83**	4 (4.8)		**81**	33 (40.7)		**80**	23 (28.8)	
N1+N2+N3	**62**	37 (59.7)		**62**	2 (3.2)		**62**	34 (54.8)		**60**	29 (47.5)	
**M stage**			**0.002**			1.000^*^			**0.037**			0.020^*^
M0	**122**	52 (42.6)		**121**	5 (4.1)		**120**	52(43.3)		**118**	39 (33.1)	
M1	**25**	19 (76.0)		**25**	1 (4.0)		**24**	16(66.7)		**24**	14 (58.3)	
**Stage**			**0.001**			1.000^*^			**0.045**			0.003^*^
I	**74**	26 (35.1)		**73**	3 (4.1)		**72**	28 (38.9)		**71**	18 (25.4)	
IS+II+III+IV	**73**	45 (61.6)		**73**	3 (4.1)		**72**	40 (55.6)		**71**	35 (49.3)	
**Vascular invasion**			0.889			0.178^*^			**0.002**			0.493^*^
No	**99**	45 (45.5)		**99**	6 (6.1)		**97**	35 (36.1)		**96**	33 (34.4)	
Yes	**43**	19 (44.2)		**43**	0 (0.0)		**42**	27 (64.3)		**42**	17 (40.5)	
**Histology**			**<0.001**			0.114^*^			**<0.001**			<0.001^*^
Seminoma	**75**	17 (22.7)		**75**	1 (1.3)		**73**	23 (31.5)		**73**	10 (13.7)	
Nonseminomatous	**74**	54 (73.0)		**73**	5 (6.8)		**73**	46 (63.0)		**71**	43 (60.6)	
**IGCCG Risk**			0.194^*^			0.452^*^			0.030^*^			0.801^*^
Low	**45**	26 (57.8)		**45**	2 (4.4)		**45**	21 (46.7)		**44**	22 (50.0)	
Intermediate	**11**	7 (63.6)		**11**	0 (0.0)		**11**	8 (72.7)		**11**	7 (63.6)	
High	**10**	9(90.0)		**10**	1(10.0)		**9**	8(88.9)		**9**	5 (55.6)	
**Recurrence**			0.108			0.542^*^			0.303			0.682^*^
No	**125**	55 (44.0)		**125**	5 (4.0)		**123**	56 (45.5)		**122**	44 (36.1)	
Yes	**17**	11 (64.7)		**17**	1 (5.9)		**17**	10 (58.8)		**17**	7 (41.2)	
**Progression**			0.468^*^			0.287^*^			0.725^*^			1.000^*^
No	**68**	40 (58.8)		**68**	2 (2.9)		**68**	37 (54.4)		**67**	31 (46.3)	
Yes	**8**	6 (75.0)		**8**	1 (12.5)		**8**	5 (62.5)		**8**	4 (50.0)	

^*^Fisher’s exact test.

### MCTs, CD147 and survival

**Figure 3:**
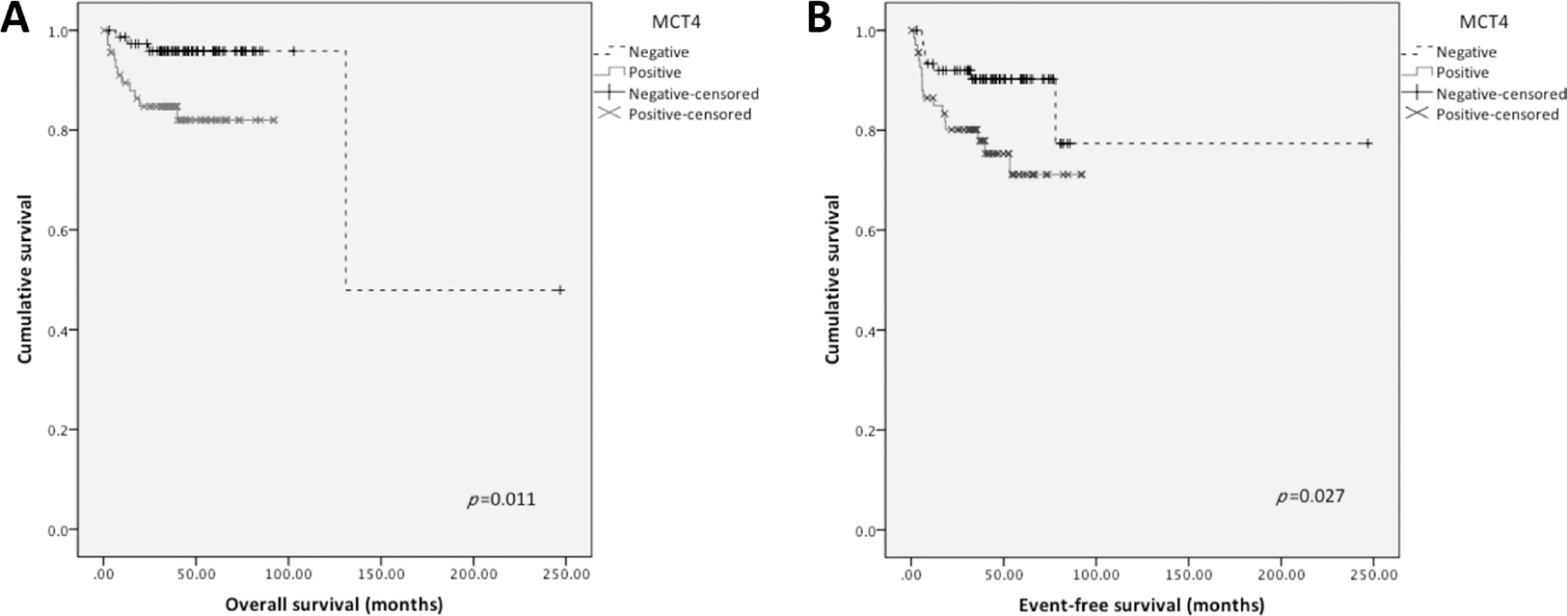
Overall and event-free survival curves of TGCTs’ patients Only significant results are shown. Continuous line refers to positive expression while interrupted line refers to negative expression. (**A**) Overall survival associated with MCT4 plasma membrane expression; (**B**) Event-free survival associated with MCT4 plasma membrane expression.

Kaplan-Meier analysis (Figure 3) revealed that MCT4 positive expression was significantly associated with shorter overall (*p* = 0.011) as well as shorter event-free survival (*p* = 0.027). Other proteins were not significantly associated with survival. None of the markers were associated with survival in multivariate analysis by Cox regression (data not shown).

### *In vitro* experiments

To further elucidate the possible contribution of MCT4 for tumor aggressiveness, *in vitro* MCT4 silencing studies were performed.

**Figure 4:**
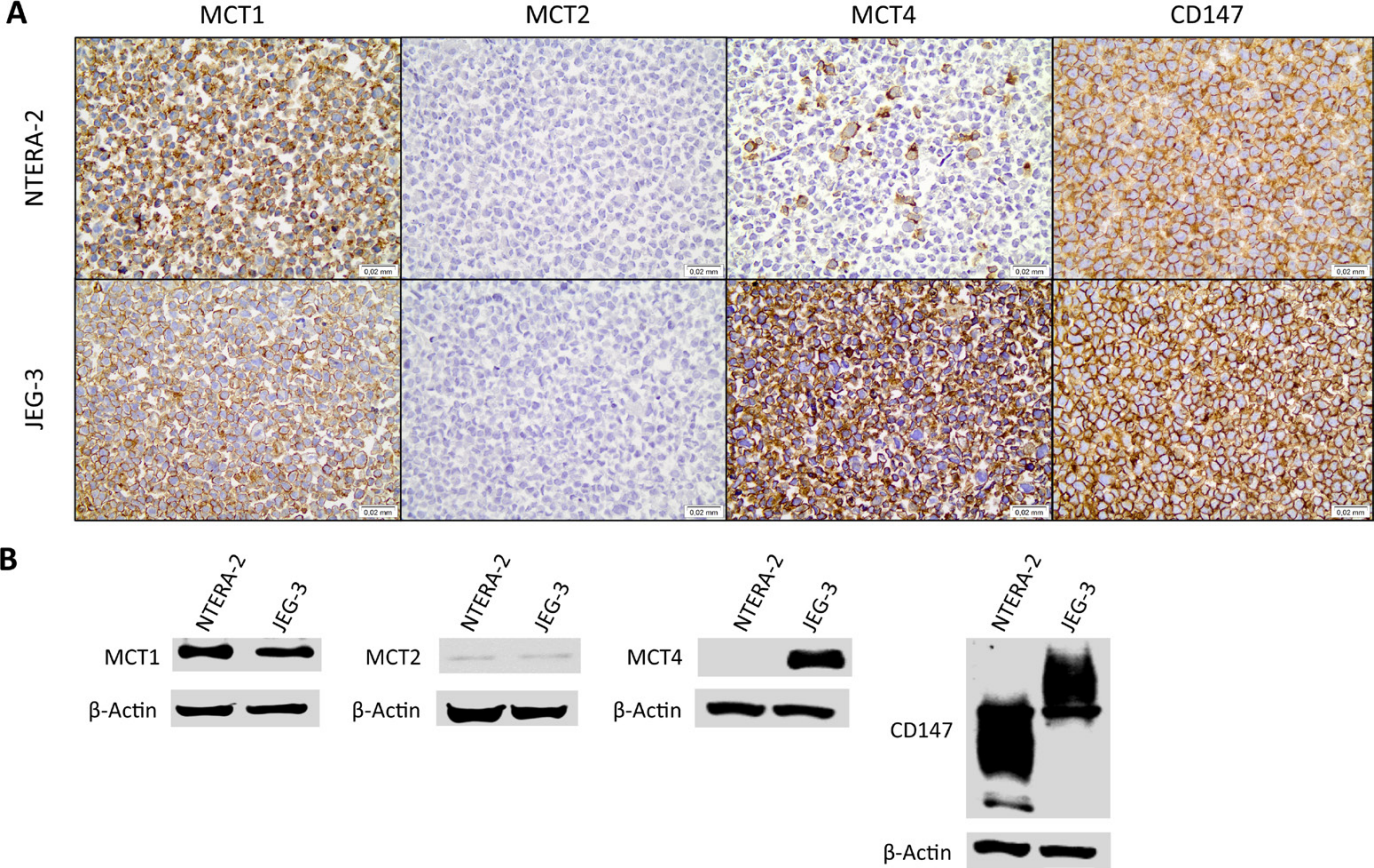
Expression of monocarboxylate transporters and the chaperone CD147 in the germ cell tumor cell lines JEG-3 and NTERA-2 (**A**) Immunocytochemical expression of MCT1, MCT2, MCT4 and CD147; (**B**) Detection of MCT1, MCT2, MCT4 and CD147 by Western blot, in cell lysates.

**Figure 5:**
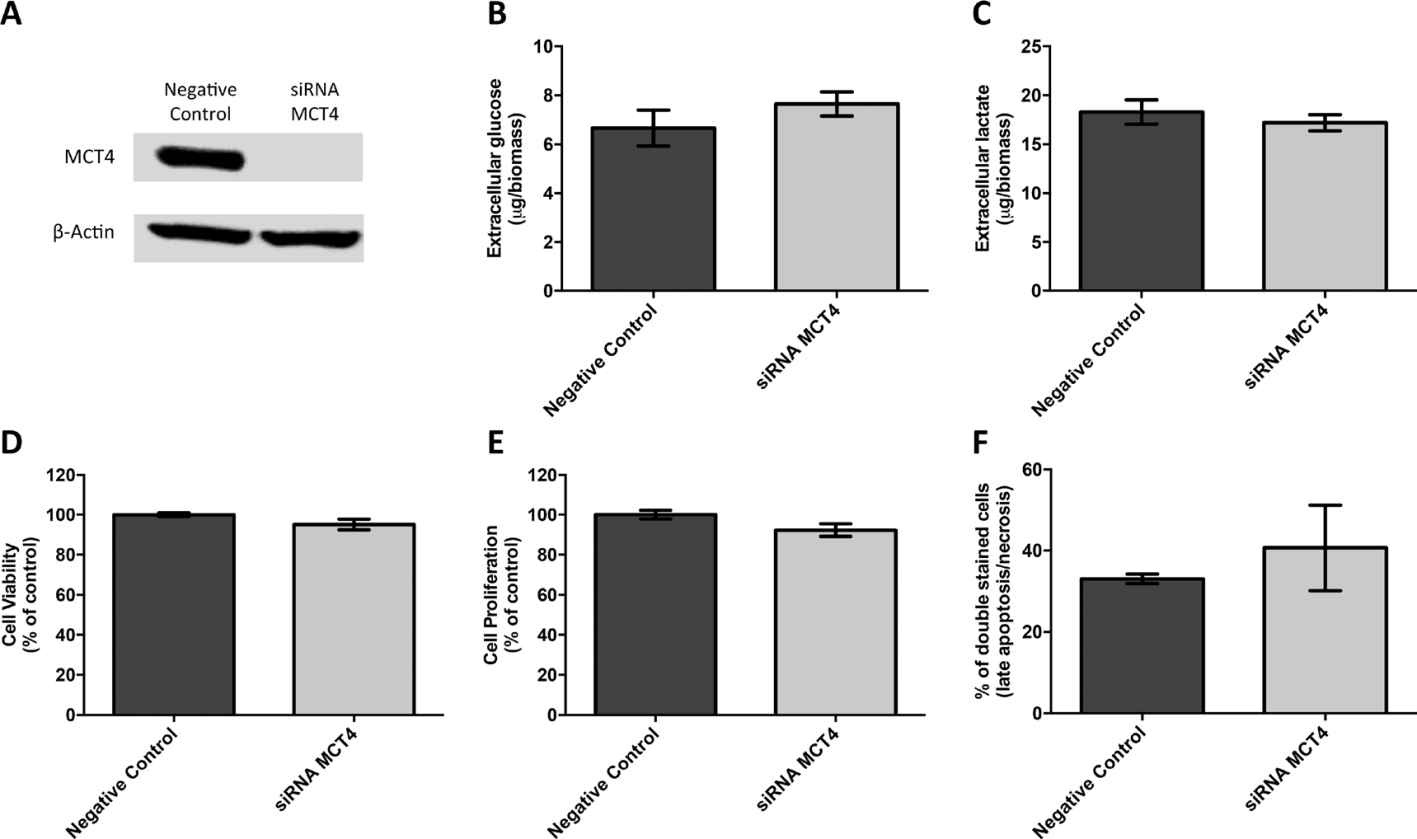
MCT4 silencing had no effect on metabolic parameters as well as viability, proliferation and death of JEG-3 cells (**A**) Western blot results for MCT4 expression after *SLC16A3* knockdown, using 10 nM siRNA, for 72 hours, showing an effective silencing of MCT4 expression. Scramble siRNA was used as negative control and β-actin was used as loading control. Extracellular amounts of glucose (**B**) and lactate (**C**) levels were quantified in culture medium obtained after 24 hours, normalized by total biomass (obtained using SRB assay) and are represented as the mean of three independent experiments in triplicate ± SEM. Cell viability was evaluated using SRB assay (**D**) while cell proliferation was evaluated using BrdU incorporation (**E**). Results were obtained after 24 hours of cell culture, normalized as the percentage of the negative control (scramble siRNA), and are represented as the mean of three independent experiments in triplicate ± SEM. Cell death was evaluated using flow cytometry analysis after Annexin V/PI staining (**F**). Results were obtained after 24 hours and are represented by the mean percentage of double stained cells (late apoptosis/necrosis) from two independent experiments ± SEM.

**Figure 6:**
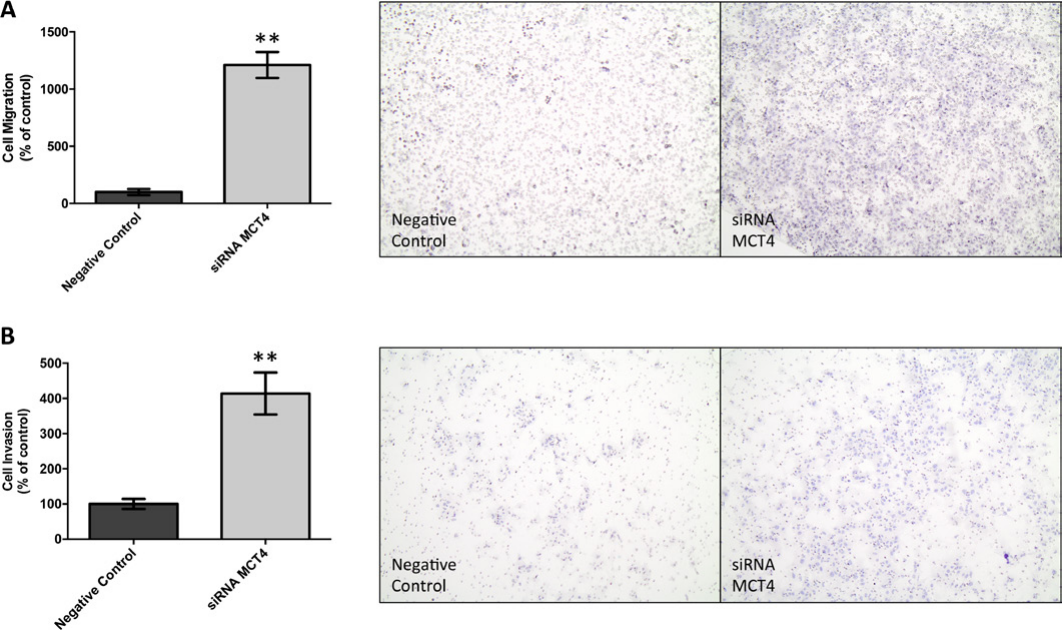
MCT4 silencing increases cell migration and invasion of JEG-3 cells Cell migration was evaluated using ThinCert Cell Cultures Inserts (Greiner Bio-One) (**A**). Results were obtained after 24 hours and are represented as the mean of two independent experiments in duplicate ± SEM (left panel). Representative images of the migration assay are shown (right panel). ^**^p = 0.0016 compared to control (scramble siRNA). Cell invasion was evaluated using Corning BioCoat Matrigel Invasion Chamber with BD Matrigel Matrix (**B**). Results were obtained after 24 hours and are represented as the mean of three independent experiments in duplicate ± SEM (left panel). Representative images of the invasion assay are shown (right panel). ^**^p = 0.0027 compared to control (scramble siRNA).

First, the germ cell tumor cell lines JEG-3 and NTERA-2 were characterized for MCT and CD147 expression. As can be seen in Figure 4A, MCT1, MCT4 and CD147 were expressed at the plasma membrane of both cell lines, although MCT4 was only detected in a small proportion of NTERA-2 cells. In opposition, MCT2 was absent from both JEG-3 and NTERA-2 cell lines. These results were validated by Western-blot (Figure 4B). Based on these results, JEG-3 cells were selected for MCT4 silencing using siRNA (Figure 5A). As depicted in Figure 5, MCT4 silencing had no effect on extracellular amounts of glucose (Figure 5B) and lactate (Figure 5C), as well as no effect of cell viability (Figure 5D), proliferation (Figure 5E) and death (Figure 5F). However, as can be seen in Figure 6, MCT4-silenced cells showed an important increase in migration (Figure 6A) and invasion (Figure 6B).

## DISCUSSION

In the present study, the expression of the metabolism related proteins MCT1, MCT2, MCT4 and CD147 was characterized in a representative sample of testicular TGCTs.

The use of the area under ROC curve to access the cutoffs for positivity allowed the definition of scores better associated with clinical events of recurrence, progression and death. The cutoffs defined were MCT1 ≥6, MCT2 ≥3, MCT4 ≥6 and CD147 ≥5. With the exception of MCT2, these scores differ from the ones used in previous studies by our group, where scores ≥3 were defined as positive [19, 38–51]. We believe that this approach for cutoff definition, which may vary among tumor type but is more illustrative of possible clinical significance, may bring results that, otherwise, may be masked by an inadequate cutoff.

TMA method demonstrated an accuracy of 79.3% for MCT1, 100% for MCT2, 76.6% for MCT4 and 79.3% for CD147. A potential limitation of this method is tumor heterogeneity, a common event in GCTs. However, in this study, cores in triplicate for each histological subtype were used, in attempt to minimize this limitation. This study is similar to others in the way that evaluated GCTs by immunohistochemistry on TMAs with cores in triplicate [32, 52]; however, those and other previous studies using TMAs to organize GCTs did not compare TMAs with whole sections [32, 52, 53]. The present study is unique since it used triplicate cores for each histological subtype and compared 31 whole sections in a sample of 149 cases on TMAs, achieving acceptable accuracy values, considering tumor heterogeneity.

MCT4 and CD147 overexpression in TGCTs, when compared to normal tissue, suggests that these tumor cells present a high lactate efflux, most probably as a result of a hyperglycolytic phenotype. These findings are in line with previous data that associate the overexpression of these proteins with hyperglycolytic phenotypes in other tumor types, being associated with malignant behavior and tumor aggressiveness [17, 19, 21, 22, 28, 54]. Although not statistically significant, the higher expression of MCT2 in normal tissue than in TGCTs is consistent with its function in lactate uptake, and with the oxidative phenotype of normal tissues [11, 14, 19, 55].

The association of MCT1, MCT4 and CD147 expression with characteristics of worse prognosis, such as nonseminomatous histology, higher stages, higher metastasis occurrence, vascular invasion and higher risk stratification, reflect the function of these proteins in lactate efflux, in maintaining the extracellular acid pH, and, as a result, in glycolytic phenotype and tumor aggressiveness. Despite the lack of studies of these proteins on TGCTs, our findings corroborate previous evidence of the association between their expression and worse prognosis in other types of cancer with glycolytic phenotype [16, 18, 54, 56, 57].

MCT4 expression was associated with lower overall and event free survival in univariate analysis, which is consistent with previous findings in other solid tumors [16, 18, 22, 54, 56–58]. However, none of the markers were associated with lower survival in multivariate analysis.

Previous studies have shown that, in general, *in vitro* MCT silencing is associated with a decrease in tumor cell aggressiveness, by means of a decrease in cell viability, proliferation, migration and invasion as well as an increase in cell death (reviewed in [24]), which would support the findings obtained in the TGCTs human samples. Herein, MCT4 silencing did not affect cell viability, proliferation or death. In fact, this is not an unexpected result, as the cell line used for MCT4 silencing also express MCT1 at the plasma membrane, which is probably providing the efflux of lactate, compensating for the lack of MCT4. Actually, accordingly, extracellular glucose and lactate levels were not affected by MCT4 silencing, an observation already made in other *in vitro* tumor model [28]. To validate this hypothesis, an attempt to a double MCT1/MCT4 silencing approach was made, however, the different timings of MCT1 *versus* MCT4 silencing using siRNA, compromised the efficiency of the double silencing (Supplementary Figure 1A and 1B). Other experimental approaches, such as stable silencing or gene editing, are warranted to further explore this hypothesis. Nevertheless, although with no influence on extracellular glucose and lactate, cell viability, proliferation and death, MCT4 silencing importantly increased cell migration and invasion. Previous studies have showed a role of MCT1 and MCT4 in tumor cell migration [23, 59–63] and invasion [62, 64], which seems to be independent from transporter activity [60]. Following this line of evidence, and considering that the result expected from MCT4 silencing is a reduction in cell migration and invasion, one can hypothesize that the increase in these parameters may result from an increase in MCT1 expression in response to MCT4 silencing. However, neither MCT1 nor CD147 expression was altered by MCT4 silencing (Supplementary Figure 1D). Additional studies are needed to further explore the specific contribution of MCT4 for tumor cell migration and invasion in germ cell tumors and others.

In conclusion, the present study validates TMA as a useful method to study MCTs and CD147 in TGCTs. Also, it demonstrates the overexpression of these proteins in tumor cells, and its association with parameters of worse prognosis and shorter survival rates. Finally, *in vitro* studies showed that individual MCT4 silencing in a cell line positive for both MCT1 and MCT4 does not decrease the main parameters of tumor aggressiveness. This study may contribute to the knowledge on the clinicopathological significance of these metabolism-related proteins and paves the way for additional studies to explore them as potential prognostic markers and possible therapeutic targets in TGCTs.

## MATERIALS AND METHODS

### Cases selection

A total of 149 cases of TGCTs were selected from the Department of Pathology of Barretos Cancer Hospital. Only samples from primary tumors, collected prior to chemotherapy and with enough formalin fixed paraffin embedded material, were selected.

The clinicopathological data collected included age, date of diagnosis, histological types, grading (when applicable), staging (TNM), presence of vascular invasion, IGCCG risk stratification, and dates of surgery, chemotherapy, recurrence and death.

Patients’ mean age was 32.3 years (ranging from 18 to 73 years) and most of them were Caucasian (80.2%). As depicted in Table 3, half of the patients presented with stage I neoplasms (50.3%) and, among stages II and III, most of the patients were low IGCCG risk (68.2%). Seminoma was the most frequent histological type (50.3%). The most frequent chemotherapy treatment used was Bleomycin, Etoposide and Cisplatin (BEP) (42.8%) followed by Etoposide and Cisplatin (EP) (9.7%) and 44.8% of the patients did no receive chemotherapy. The median follow up was 40.3 months and the majority of patients were alive and free of disease at the end of the study (85.9%).

**Table 3:** Clinicopathological parameters of the patients

	*n* (%)
**Histological type**	
Yolk sac	1 (0.7)
Choriocarcinoma	2 (1.3)
Embryonal Carcinoma	8 (5.4)
Immature Teratoma (grade I)	2 (1.3)
Seminoma	75 (50.3)
Mixed GCT	41 (27.5)
**Stage at diagnosis**	
I	74 (50.3)
II	35 (23.8)
III	31 (21.1)
IV	0 (0.0)
IS	7 (4.8)
**IGCCG Risk**	
Low	45 (68.2)
Intermediate	11 (16.7)
High	10 (15.2)
**Chemotherapy**	
BEP	62 (42.8)
EP	14 (9.7)
Other	4 (2.8)
No Chemotherapy	65 (44.8)
**Status – post treatment**	
Alive and disease free	128 (85.9)
Alive and in treatment	5 (3.4)
Cancer related death	13 (8.7)
Death from other causes	3 (2.0)

The present study was approved by the Local Ethic Committee (number 541235).

### TMA construction and validation

All the cases were reviewed by an experienced pathologist (ECAS) for diagnostic confirmation and demarcation of tumor areas for TMA cores. Each tumor histological subtype and corresponding normal tissues were selected (when available and sufficient) for triplicate cores of 1.0 mm. Tissue cores of liver, kidney and placenta were used as controls for TMA orientation.

For TMA validation, 31 cases were randomly selected based on sample calculation, with a confidence interval of 95%. For these 31 cases, immunohistochemistry was performed on both TMA and whole sections, and the results compared.

### Immunohistochemistry

Immunohistochemistry was performed on TMA samples and whole sections, according to manufacturer’s instructions and as previously described [19]. MCT1 immunohistochemistry was performed using a polymer system (UltraVision LP Detection System: HRP Polymer, Lab Vision Corporation, Fremont, CA). MCT2 and MCT4 immunohistochemistry was performed using the streptavidin-biotin-peroxidase complex principle (Ultravision Detection System: Anti-polyvalent, HRP, Lab Vision Corporation, Fremont, CA). For CD147, the avidin-biotin-peroxidase complex method (VECTASTAIN Elite ABC kit R.T.U., Vector, Burlingame, CA) was used. For details on each antibody, see Table 4. Slides were counterstained with hematoxylin and permanently mounted. Colon carcinoma tissue was used as positive control for all antibodies.

**Table 4:** Detailed aspects for each antibody used in immunohistochemistry

Protein	Antigen retrieval	Antibody	Antibody dilution and incubation time
**MCT1**	Citrate buffer (0.01 M, pH = 6), 98° C, 20′	AB3538P Chemicon International	1:1000, overnight
**MCT2**	Citrate buffer (0.01 M, pH = 6), 98° C, 20	sc-50322 Santa Cruz Biotechnology	1:200, 2 hours
**MCT4**	Citrate buffer (0.01 M, pH = 6), 98° C, 20′	sc-50329 Santa Cruz Biotechnology	1:500, 2 hours
**CD147**	EDTA (1 mM, pH = 8), 98° C, 20’	sc-71038 Santa Cruz Biotechnology	1:400, overnight

Plasma membrane expression was evaluated semi-quantitatively for extension and semi-qualitatively for intensity. For extension, the grading was: 0: no positive cells; 1: <5% of positive cells; 2: 5–50% of positive cells; 3: >50% of positive cells. Staining intensity was graded as follows: 0: negative; 1: weak; 2: moderate; 3: intense. The final score was obtained by the sum of both grades (minimum 0 and maximum 6).

TMA and whole sections immunostains were evaluated by two experienced pathologists independently (ECAS and CS-N). Discordant cases were reviewed and scored in consensus.

### Cell lines and cell culture

The germ cell tumor cell lines NTERA-2 and JEG-3 were used in this study. Both cell lines were used for protein characterization using immunocytochemistry and Western Blotting and JEG-3 cell line was used for *in vitro* studies. The cell lines were maintained in Dulbecco’s Modified Eagle’s Medium (DMEM 1×, High Glucose, Gibco, Invitrogen) supplemented with 10% fetal bovine serum, FBS (Gibco, Invitrogen) and 1% penicillin-streptomycin solution (Gibco, Invitrogen) at 37° C and 5% CO_2_. The cells were authenticated by genotyping (panel of 8 short tandem repeat loci) as previously described [65], and validated as mycoplasma negative.

### Paraffin cytoblock and immunocytochemistry

Paraffin cytoblocks were made from concentrated cell suspensions by centrifuging fresh cell suspensions at 1500 rpm for 5 min. Cell pellets were incubated with formaldehyde 10% for 1 hour and recentrifuged. Fixed pellets were included in agarose 2% PBS 1× at 60° C, homogenized and centrifuged at 13000 rpm for 2 min. Solid agarose cell pellets were cuted in two halfs for processing and paraffin inclusion. Immunocytochemistry for MCT1, MCT2, MCT4 and CD147 was performed in 4 µm cytoblock sections, as described for immunohistochemistry for human samples.

### Western blotting

NTERA-2 and JEG-3 GCTs cell lines were grown to 70–80% confluence, washed in cold PBS and then scrapped and homogenized in lysis buffer containing 50 mM Tris (pH = 7.5), 150 mM NaCl, 0.1 mM EDTA, 1% Triton X-100 and 1% NP40, supplemented with 1:10 protease inhibitors (cOmplete Protease Inhibitor Cocktail, Roche) according to the manufacturer’s instructions. The lysates were incubated on ice for 15 min and then centrifuged at 13000 rpm for 15 min at 4° C. The supernatants were collected and protein concentration was determined using the Bradford assay (Bio-Rad Protein Assay kit, Bio-Rad). Proteins were dissolved in sample buffer (Laemmli 2× Concentrate, Sigma-Aldrich) and boiled for 5 min at 95°C. Aliquots of 20 µg of total protein were separated on 10% polyacrylamide gel by SDS-PAGE and transferred onto a nitrocellulose membrane (Protran, Amersham, GE Healthcare) in 25 mM Tris-base/glycine buffer by TransBlot Turbo transfer (Bio-Rad). Membranes were blocked with 5% non-fat dry milk in TBS/0.1% Tween (TBS-T; pH = 7.6) for 1 hour at room temperature. After overnight incubation at 4° C with the primary antibodies (dilution in TBS-T, 5% BSA) for MCT1 (1:200 dilution, H-1, sc-365501, Santa Cruz Biotechnology), MCT2 (1:1200 dilution, ab81262, Abcam), MCT4 (1:2000 dilution, H-90, sc-50329, Santa Cruz Biotechnology) and CD147 (1:250 dilution, 1.BB.218, sc-71038, Santa Cruz Biotechnology), membranes were washed in TBS-T and incubated with anti-rabbit (sc-2020, Santa Cruz Biotechnology) or anti-mouse (sc-2031, Santa Cruz Biotechnology) secondary antibodies coupled to horseradish peroxidase (1:5000 dilution in TBS-T, 5% non-fat dry milk). The bound antibodies were visualized by chemiluminescence (Supersignal West Femto kit, Pierce) in ImageQuant LAS 4000 mini (GE Healthcare). β-Actin was used as loading control at 1:2000 dilution (8H10D10, Cell Signaling Technology).

### RNA interference and transfection

Silencing experiments were performed using 10 nM of Silencer Select Validated siRNA for MCT1 (*SLC16A1* siRNA, s580, Ambion) and MCT4 (*SLC16A3* siRNA, s17417, Ambion), as well as nontargeting control siRNA (Silencer Select Negative Control No.1 siRNA, 4390843, Ambion), using 1 μl/ml of Lipofectamine RNAiMAX (13778–075, Invitrogen), according to the manufacturer’s instructions. For MCT1, total silencing was only observed after 1 day, with the reestablishment of the MCT1 expression at day 2 (48 hours, confirmed by Western Blot, Supplementary Figure 1A). For MCT4, total silencing was only observed after 72 hours, being maintained for at least 2 additional days (120 hours, confirmed by Western Blot, Supplementary Figure 1B and 1C). The cells were plated at day 3 after silencing, when the experiments begin.

### Cell biomass analysis

The effect of MCT4 knockdown on total biomass was measured by the Sulforhodamine B assay (TOX-6, Sigma-Aldrich), according to the manufacturer’s instructions. For that, 1.0 × 10^4^ cells/well of silenced and negative control cells were plated in 96-wells plates in complete DMEM medium culture. Total biomass was quantified after 24 hours. Results are represented as percentage of total biomass normalized for the control condition, of three independent experiments, in triplicate.

### Metabolism assay (extracellular glucose and lactate measurements)

The metabolic behavior of MCT4 knockdown JEG-3 cell line was determined by analyzing the extracellular amounts of glucose and lactate. For that, 1.0 × 10^4^ cells/well of silenced and negative control cells were plated in 96-wells plates in complete DMEM medium culture. Glucose and lactate measurement were performed after 24 hours, with commercial kits (Cayman Chemical and SpinReact, respectively), according to the manufacturer’s instructions. Results are expressed as total μg/total biomass of three independent experiments, in triplicate.

### Cell proliferation assay

The proliferation capacity after silencing of MCT4 on JEG-3 cell line was assessed by bromodeoxyuridine incorporation. For that, 1.0 × 10^4^ cells/well of silenced and negative control cells were plated in 96-wells plates in complete DMEM medium culture. To determine the percentage of proliferation, cells were incubated with 10 μM bromodeoxyuridine for 24 hours and its incorporation was assessed at 370 nm (λ_ref_ = 492 nm), according to the manufacturer’s protocol (Cell Proliferation ELISA, BrdU, Roche). Results are represented as percentage of proliferation normalized for the control condition, of at least three independent experiments, in triplicate.

### Cell death assay

Apoptotic/necrotic cell population was determined by Annexin V-FITC Apoptosis Detection Kit I (BD Biosciences) according to the manufacturer’s instructions. For that, 4.0 × 10^5^ cells/well of silenced and negative control cells were plated in 6-wells plates in complete DMEM medium culture. After 24 hours, cells were collected and Annexin V/PI staining was performed with incubation for 15 min at room temperature. The percentage of cell death (double staining) was assessed by flow cytometry (BD Accuri C6, BD Biosciences), with a total of 20000 events collected for each condition. Results were analyzed using the BD Accuri C6 Software (version 1.0.264.21) and are represented as percentage of cell death normalized for the control condition, of two independent experiments.

### Migration assay

Migration capacity of JEG-3 cell line was measured using ThinCert Cell Cultures Inserts (Greiner Bio-One), according to the manufacturer’s instructions. In brief, 1.0 × 10^5^ cells/well of silenced and negative control cells were plated in 24-well transwell inserts in DMEM, 0% FBS. DMEM supplemented with 10% FBS was used as chemoattractant. Cells were allowed to migrate for 24 hours. Then, the cells that did not migrate were removed and migrating cells were fixed with methanol and stained with hematoxylin and eosin for 10 min. Membranes were photographed in Olympus BX43 (×10 magnification), and migrating cells were counted using the OpenCFU software (version 3.8) [66]. Results are represented as percentage of cell migration normalized for the control condition, of two independent experiments in duplicate.

### Invasion assay

Invasion capacity of JEG-3 cell line was measured using Corning BioCoat Matrigel Invasion Chamber with BD Matrigel Matrix (Corning), as indicated by the manufacturer. In brief, 2.5 × 10^5^ cells/well of silenced and negative control cells were plated in the matrigel-coated 24-well transwell inserts in DMEM, 0% FBS. DMEM supplemented with 10% FBS was used as chemoattractant. Cells were allowed to invade for 24 hours. Then, the noninvading cells were removed and invading cells were fixed with methanol and stained with hematoxylin and eosin for 10 min. Membranes were photographed in Olympus BX43 (×10 magnification), and invading cells were counted using the OpenCFU software (version 3.8) [66]. Results are represented as percentage of cell invasion normalized for the control condition, of at least three independent experiments in duplicate.

### Statistical analysis

Results obtained with the human samples were analyzed using IBM SPSS Statistics software (version 23.0, IBM Company, Armonk, NY). ROC curve was used to define final score’s cutoff for positivity, based on the area under the curve. Frequency of protein expression and comparison with clinicopathological data were analyzed using McNemar’s test, Pearson’s chi-square test and Fisher’s exact test, according to the sample’s characteristics. The agreement between TMA and whole sections was evaluated by the accuracy of the method. Overall and event-free survival curves were constructed using Kaplan-Meier’s method and the data compared with log-rank test. Cox regression model was used for multivariate analysis. *In vitro* results were analyzed using GraphPad Prism software (version 6.0, GraphPad Software, Inc., La Jolla, CA), with Student’s *t* test (Welch’s correction was used in case of unequal variances). For all tests, the level of significance was established as 5% (significant results if *p* < 0.05).

## SUPPLEMENTARY MATERIALS FIGURE


