# Supplementary material for: The clinicopathological significance of monocarboxylate transporters in testicular germ cell tumors
Source: Oncotarget. 2018 Apr 17;9(29):20386–98. doi: 10.18632/oncotarget.24910 (PMC5945514; doi:10.18632/oncotarget.24910)
Supplement: Supplementary file 1 [file oncotarget-09-20386-s001.pdf]

The clinicopathological significance of monocarboxylate transporters in testicular germ cell tumors

SUPPLEMENTARY MATERIALS

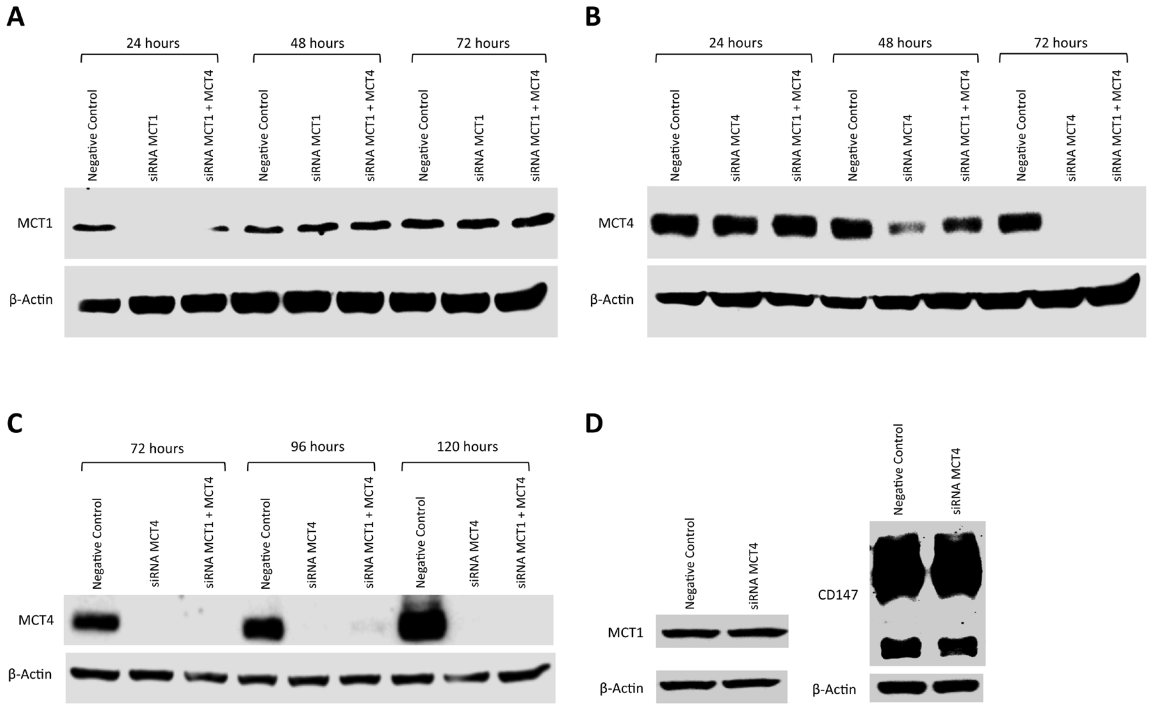

Supplementary Figure 1: MCT1 and MCT4 show different silencing kinetics in JEG-3 cells (**A**) Western blot results for MCT1 expression after *SLC16A1* individual knockdown and *SLC16A1+SLC16A3* double knockdown, using 10 nM siRNA, for 24, 48 and 72 hours, showing an effective silencing of MCT1 expression at 24 hours. Scramble siRNA was used as negative control and β-actin was used as loading control. (**B**) Western blot results for MCT4 expression after *SLC16A3* individual knockdown and *SLC16A1+SLC16A3* double knockdown, using 10 nM siRNA, for 24, 48 and 72 hours, showing an effective silencing of MCT4 expression at 72 hours. Scramble siRNA was used as negative control and β-actin was used as loading control. (**C**) Western blot results for MCT4 expression after *SLC16A3* individual knockdown and *SLC16A1+SLC16A3* double knockdown, using 10 nM siRNA, for 72, 96 and 120 hours, showing that the silencing observed at 72 hours lasts at least until 120 hours. Scramble siRNA was used as negative control and β-actin was used as loading control. (**D**) Representative Western blot results for MCT1 and CD147 expression after *SLC16A3* knockdown, using 10 nM siRNA, for 72 hours, showing similar expression levels after MCT4 silencing. Scramble siRNA was used as negative control and β-actin was used as loading control.
